# Phasic Burst Stimulation: A Closed-Loop Approach to Tuning Deep Brain Stimulation Parameters for Parkinson’s Disease

**DOI:** 10.1371/journal.pcbi.1005011

**Published:** 2016-07-14

**Authors:** Abbey B. Holt, Dan Wilson, Max Shinn, Jeff Moehlis, Theoden I. Netoff

**Affiliations:** 1 Graduate Program in Neuroscience, University of Minnesota, Minneapolis, Minnesota, United States of America; 2 Department of Mechanical Engineering, University of California, Santa Barbara, California, United States of America; 3 Department of Neuroscience, University of Minnesota, Minneapolis, Minnesota, United States of America; 4 Department of Biomedical Engineering, University of Minnesota, Minneapolis, Minnesota, United States of America; Indiana University, UNITED STATES

## Abstract

We propose a novel, closed-loop approach to tuning deep brain stimulation (DBS) for Parkinson’s disease (PD). The approach, termed Phasic Burst Stimulation (PhaBS), applies a burst of stimulus pulses over a range of phases predicted to disrupt pathological oscillations seen in PD. Stimulation parameters are optimized based on phase response curves (PRCs), which would be measured from each patient. This approach is tested in a computational model of PD with an emergent population oscillation. We show that the stimulus phase can be optimized using the PRC, and that PhaBS is more effective at suppressing the pathological oscillation than a single phasic stimulus pulse. PhaBS provides a closed-loop approach to DBS that can be optimized for each patient.

## Author Summary

Deep brain stimulation (DBS) is effective at treating motor symptoms of patients with medication-refractory Parkinson’s disease (PD). Currently, high frequency stimulation (>100 Hz) is tuned for each patient using a trial-and-error process. A systematic approach to tuning stimulation parameters based on patient physiology has the potential to improve patient quality of life. We present a novel closed-loop approach to DBS, termed Phasic Burst Stimulation, where the timing of stimulus pulses is optimized using an algorithm based on patient physiology. A closed-loop approach has the potential to improve therapeutic efficacy, extend battery lifetime by decreasing power consumption, decrease negative side effects by more selectively stimulating, and adjust as motor symptoms fluctuate.

## Introduction

Deep brain stimulation (DBS) is a neuromodulation therapy effective at treating motor symptoms of medication-refractory Parkinson’s disease (PD). Tuning stimulation parameters is currently done using a time intensive trial-and-error process [1]. Implantable DBS devices have been developed for research that can simultaneously deliver stimulation while recording the neural response [2]. Soon these devices will enable a closed-loop approach to setting stimulation parameters. A closed-loop tuning algorithm has the potential to reduce time spent in the clinic setting stimulation parameters and may result in more robust and effective tuning. Furthermore, a closed-loop device can continuously tune parameters to maintain maximal efficacy as motor symptoms fluctuate.

Dynamic changes in the basal ganglia network are thought to lead to motor symptoms of PD. A loss of dopaminergic inputs results in changes in firing rates and patterns of neurons within the basal ganglia. The emergence of synchronous activity, particularly in the beta range (12–35 Hz), is hypothesized to give rise to motor symptoms of PD [3–8]. While the role of beta oscillations is debated, therapeutic DBS has been shown to disrupt the oscillation [9–11]. Furthermore, closed-loop adaptive stimulation approaches, where high frequency stimulation is turned on when the amplitude of the beta oscillation is high, have been shown to produce greater improvement in akinetic/rigid motor symptoms while using less battery power [12]. This suggests that the beta oscillation may be a good biomarker for closed-loop stimulation.

Delivering electrical pulses at a specific phase of the ongoing pathological oscillation has the potential to more efficiently disrupt this activity than the high frequency periodic stimulation used currently. High frequency DBS, >100 Hz, is more therapeutically effective than low frequency [13] or random stimulation [14]. This may be because periodic stimulation at certain frequencies results in more stimulus pulses occurring at phases that desynchronize neurons generating the beta oscillation [15, 16]. Therefore, applying closed-loop stimulation where stimulus pulses are locked to a specific phase of the oscillation may be more effective at disrupting the pathological beta oscillation than high frequency open-loop stimulation. In essential tremor patients, phase dependent modulation of the tremor amplitude is seen when stimulus pulses to the thalamus are locked to specific phases of the tremor [3, 15]. There has not yet to our knowledge been an experiment that delivers stimulation pulses phase locked to the beta oscillation in the basal ganglia.

Provided a real-time estimation of the phase of a pathological oscillation, it is necessary to develop an algorithm that can determine the optimal phase to deliver the stimulus. In order to identify this optimal phase we propose to use a measure called the phase response curve (PRC). A PRC is a simple measure that describes how the phase of the oscillation is affected by the phase at which a perturbation, such as an external stimulus, is delivered. The PRC can be used to predict conditions in which coupled oscillators, such as periodically firing neurons, will synchronize or desynchronize [17]. PRCs have been used to predict stimulus parameters, such as frequency and amplitude [18] as well as non-pulsatile stimulus shapes [19] to synchronize or desynchronize model neurons.

The oscillatory activity seen in Parkinson’s disease is seen in the neural field activity. We have previously shown that the PRC measured from a population oscillation in a computational model of the subthalamopallidal network can be used to predict the effect of stimulation frequency on the oscillation amplitude [20]. This model, developed by Hahn & McIntyre [21], produces an emergent 34 Hz population oscillation in the PD state. In this paper we show it is possible to accurately predict the effects of phasic stimulation on the amplitude of the population oscillation in the Hahn & McIntyre model using the PRC.

While a single pulse delivered at the optimal phase may suppress oscillations with high efficacy, we hypothesize that delivering multiple pulses over a range of phases may be even more effective. Here we propose a phasic burst stimulation protocol (PhaBS) optimized using the PRC. The PRC can be used to determine both the stimulus phase and burst duration to suppress the oscillation.

This paper demonstrates three points: 1) there is a phase dependent effect of stimulation on the population oscillation, 2) the population PRC can be used to predict these effects, and 3) a burst of stimulus pulses over a range of phases is more effective at disrupting the oscillation than a single stimulus pulse. While the focus of this paper is on designing stimulation to suppress oscillations in this particular computational model, this approach may be generalized to other population oscillations.

## Methods

### Computational Model

**Fig 1:**
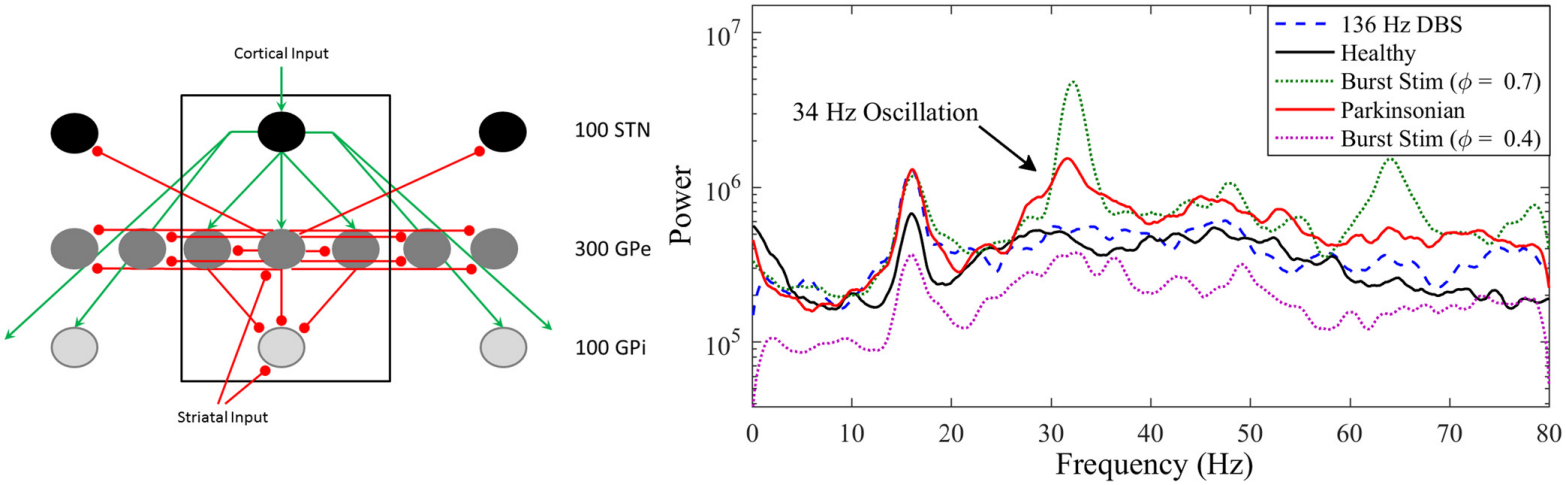
Hahn and McIntyre model displays an emergent 34 Hz parkinsonian population oscillation. Left: Connectivity of the Hahn and McIntyre model consisting of 300 conductance based neurons in the globus pallidus external (GPe), 100 neurons in the subthalamic nucleus (STN), and 100 neurons in the globus pallidus internal (GPi). Right: Power spectrum showing the emergence of a 34 Hz pathological oscillation in the parkinsonian state (red), which is not present in the healthy state (black) and is suppressed with 136 Hz DBS (dotted). The peak at 16 Hz is caused by the cortical input into the network model.

In this paper we test the effects of closed-loop phasic stimulation on the amplitude of a population oscillation in a computational model of the subthalamopallidal network developed by Hahn & McIntyre [21]. We chose this model because it exhibits an emergent 34 Hz population oscillation in the PD state [20], shown in Fig 1, that is modulated with stimulation to the subthalamic nucleus (STN).

The Hahn & McIntyre model consists of 500 single compartment conductance-based neurons: 100 globus pallidus internal neurons (GPi), 100 STN neurons, and 300 globus pallidus external (GPe) neurons. Excitatory cortical synaptic drive to STN is simulated as a 16 Hz stochastic bursting input. Inhibitory striatal synaptic drive is delivered to GPi and GPe. Parameters for the parkinsonian state were fit using *in vivo* microelectrode recordings from non-human primates [22–24]. The model was tuned to replicate the mean firing rates and bursting rate within each population (STN, GPe, and GPi) as well as their shifts in the parkinsonian state using a least squares error optimization [21].

While the original Hahn & McIntyre paper focused on the bursting rates of different populations, we previously demonstrated that a 34 Hz population oscillation emerges in the parkinsonian state due to neurons in the STN and GPe resonating better with each other [20]. This oscillation can be seen in the power spectrum calculated from the summation of phases of the GPe population (Fig 1). Importantly, this population oscillation is reduced with high frequency stimulation to the STN, a common target for DBS in patients with PD. While this pathological oscillation occurs at a higher frequency than commonly seen in PD patients, it offers a biomarker not present in the healthy state that is modulated by the simulated DBS therapy (unlike the 16 Hz input from the cortex). Therefore, the Hahn & McIntyre model provides a platform to test the effects of optimized closed-loop stimulation on an emergent pathological oscillation.

While there are many effects of STN DBS, the focus of the Hahn & McIntyre model is on targeting efferent activity. In the model, DBS is simulated by activating neurons in the efferent target. To model antidromic effects of the axonally generated action potentials, a subthreshold current injection is applied to STN neurons. The amplitude of the direct current injections was set to 1/10th of that used in the original paper in order to modulate the phase of the population oscillation rather than resetting the phase.

As with any computational model, there are limitations to the Hahn & McIntyre model. First, while the neurons in the model are coupled based on anatomy, the actual wiring arrangements in the basal ganglia are much more complex. Second, heterogeneity within the cells in the model is caused by random input to identical model neurons, while neurons in the basal ganglia have a lot of heterogeneity in their firing rates and PRCs [25]. Third, there is a complex topology to any neural network, which is not represented in this model. Fourth, the effects of stimulation are a simplification. For computational efficiency, dendritic arbors are not simulated and therefore stimulation is applied as a direct current injection to the cells. Furthermore, as there is no cortical population included in the model, antidromic activation of cortical neurons as a result of STN DBS is not included [26]. While this is not an accurate representation of how DBS affects the neural tissue [21], the model does the best job at modeling the effects of DBS on efferent targets, where our analysis focuses.

#### Closed-loop phasic stimulation

To test closed-loop phasic stimulation, a real-time estimation of the population phase is necessary to determine when to apply the stimulus. The phase was estimated using a time-weighted Fourier transform of the spikes times occurring in the previous *T* = 400*ms* at frequency, *ω* = 34 Hz:

$$\begin{array}{c} X\left(f,t\right)=\sum_{k=1}^{N_{S}}e^{(S_{k}-t)/\tau }e^{-2\pi jfS_{k}}, \end{array}$$ (1)

where *S*_*k*_ is the time of the *k*^*th*^ spike of the GPe population, *N*_*S*_ is the number of spikes in GPe from time *t* − 400 ms until the present time, *t*, and *τ* = 3 ms defines the time constant of the time-weighting of the fit.

The instantaneous population phase at time t, *ϕ*(*t*) can be determined as follows:

$$\begin{array}{c} \phi \left(t\right)=∠(\sum_{f=f_{\text{min}}}^{f_{\text{max}}}X\left(f,t\right)e^{2\pi jtf}), \end{array}$$ (2)

where *f* is the frequency range of the beta oscillation, *f*_*min*_ = 30 Hz and *f*_*max*_ = 36 Hz.

Stimulation was applied at a specified delay after the midpoint of the oscillation was detected, i.e. where *ϕ*(*t*) = 0. 100 second simulations were run for 10 delay values. Depending on the simulation being tested, either a single stimulus pulse or a bursts of three equally spaced stimulus pulses were applied. The power of the pathological beta oscillation (31–36 Hz) compared to the baseline gamma (60–64 Hz) was measured across each cycle.

### Predicting Desynchronizing Stimulation Phase

#### Phase response curve theory

We use phase response curve (PRC) theory to predict how phasic stimulation locked to an oscillation will desynchronize neurons. The phase advance, Δ, that occurs from a stimulus applied at phase *ϕ* is characterized by a function *Z*(*ϕ*), which can be measured directly from a neuron or population of neurons. When stimulation of an oscillator is periodic, the PRC can be used to predict the phase of the stimulus on the next cycle, *ϕ*_*i*+1_, from the phase on the current cycle *ϕ*_*i*_ via a map, *ϕ*_*i*+1_ = *ϕ*_*i*_ + *Z*(*ϕ*_*i*_).

**Fig 2:**
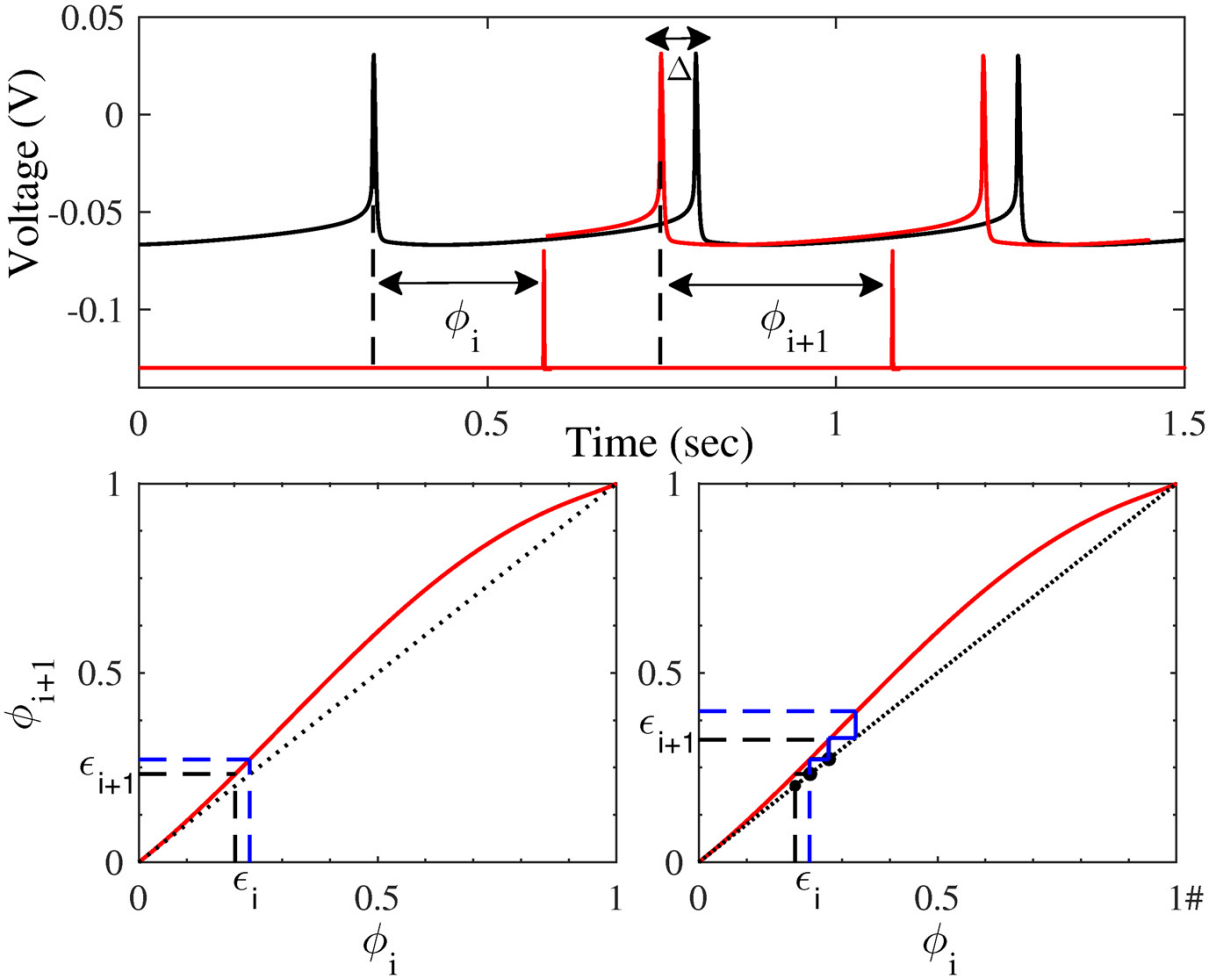
Phase response curves can be used to predict synchronization properties of a periodic stimulus. Top: Current pulses for a periodic stimulus (red pulses) can be applied to a periodically firing neuron (black). The stimulus pulse is applied at a specific phase (*ϕ*_*i*_) in the cycle of the neuron. This results in a change in the timing of the next action potential (red voltage trace). The difference in spike timing is measured as the spike time advance (Δ*ϕ*_*i*_). The stimulus will now fall at a different phase (*ϕ*_*i*+1_) on the next cycle. Bottom left: The phase of the stimulus on the next cycle can be predicted from the PRC map (black; PRC added to the red line of identity). Given the phase of the current stimulus pulse (*ϕ*_*i*_), the map can be used to predict the phase on the next cycle (*ϕ*_*i*+1_). The slope of the PRC map at the stimulus phase affects how two neurons (black and blue) will synchronize or desynchronize. Here, where the slope of the PRC map is greater than the diagonal (red line), the two neurons starting at some small distance in phase apart (*ϵ*_*i*_) are further apart on the next cycle, *ϵ*_*i*+1_ > *ϵ*_*i*_. Bottom right: The PRC map can be used to predict the stimulus phase on the next cycle (*ϕ*_*i*+1_) for a burst of three stimulus pulses. Here each stimulus pulse is applied at a phase where the slope of the PRC map is positive. This results in a greater separation of the two neurons on the next cycle (*ϵ*_*i*+1_) than when a single pulse was used (bottom left).

PRC theory can be described using two periodically neurons starting relatively close together in phase. The distance in phase between the two neurons on the next cycle can be determined from the PRC and their distance, *ϵ*, on the current cycle *ϵ*_*i*+1_ = *ϵ*_*i*_ × (1 + *Z*′(*ϕ*_*i*_)). If the absolute value of the slope of the map at the phase the stimulus is delivered is greater than one, then the distance between the neurons will grow, as shown in Fig 2. Application of the stimulus over several cycles at these phases will cause the neurons’ phases to diverge and desynchronize. The optimal stimulus phase to desynchronize the neurons with a single pulse per cycle of the oscillation is the phase at which the map has the steepest positive slope. In this description, the oscillators are individual neurons. However, in the Hahn & McIntyre model, we have a population oscillation, where the populations of neurons are the oscillators.

A burst of stimulation pulses applied over a range of phases where the absolute value of the slope of the map is greater than one will increase the phase difference between the neurons on the next cycle over a single pulse. Assuming no interaction between stimuli, this is shown in Fig 2. We hypothesize that burst stimulation will be more effective at desynchronizing a population of neurons than pulsatile stimulation.

#### Estimation of a PRC from a population oscillation

In a previous paper we describe how to estimate a PRC from a population oscillation [20] where it is described in full. This method will be described briefly here. The Fourier coefficient, *c*_*β*_ was estimated directly from the spike time data output from the Hahn & McIntyre model. The Fourier coefficient is a complex number represented as *c*_*β*_ = *Ae*^−*jϕ*^. The phase can be determined by taking the angle, ∠, of the Fourier coefficient. A Fourier coefficient was fit to a 94 msec window (approximately three cycles of the oscillation) before, $c_{i}^{prestim}$, and after, $c_{i}^{poststim}$, the stimulus *i*. The phase change can be determined by taking the difference between the phase angle estimated from the time immediately after the stimulus and the phase angle estimated immediately before the stimulus, $\Delta \phi_{i}^{\beta }=∠c_{i}^{prestim}-∠c_{i}^{poststim}$. The mean and standard deviation of the phase advance for each phase bin was estimated by fitting a wrapped normal distribution.

#### Predicting the effect of stimulation on synchrony with the PRC

Using the PRC we are able to predict the effect of phasic stimulation on the amplitude of a population oscillation. The precise phase divergence can be calculated, taking into account the stimulus pulse interval:

$$\begin{array}{c} \epsilon_{i+1}=\epsilon_{i}\left(1+Z_{1}^{\prime }\left(\phi_{i}\right)\right)\left(1+Z_{2}^{\prime }\left(\phi_{i}+Z_{1}\left(\phi_{i}\right)+\delta \right)\right)\left(1+Z_{3}^{\prime }\left(\phi_{i}+Z_{1}\left(\phi_{i}\right)+Z_{2}\left(\phi_{i}+Z_{1}\left(\phi_{i}\right)+\delta \right)+2\delta \right)\right), \end{array}$$ (3)

where *ϵ*_*i*+1_ is the phase difference between two neurons after the three stimuli; *ϵ*_*i*_ is the time difference between two neurons on their *i*^th^ spike; *δ* is the inter-stimulus-interval; and *Z*_1_, *Z*_2_, and *Z*_3_ are phase response functions associated with the first, second, and third stimulus pulse, respectively. These functions may differ from each other if they explicitly account for higher order resetting characteristics resulting from pulsing history. While this equation solves for effect on synchrony after three stimulus pulses, the equation can be generalized to any number of pulses.

Fig 2 assumes the effects of each stimulus input have dissipated by the time the next stimulus is applied, and that each stimulus pulse has the same effect on the phase of the oscillation. In this case, where only first order effects are considered, *Z*_1_, *Z*_2_, and *Z*_3_ are identical. However, there may be interactions between stimuli, or higher order effects [27]. In this paper, we look at both the first order PRC, measured by applying a single subthreshold stimulus pulse at 2 Hz, as well as a PRC measured using a burst of 3 stimulus pulses, as was used for PhaBS simulations, to address higher order effects.

## Results

**Fig 3:**
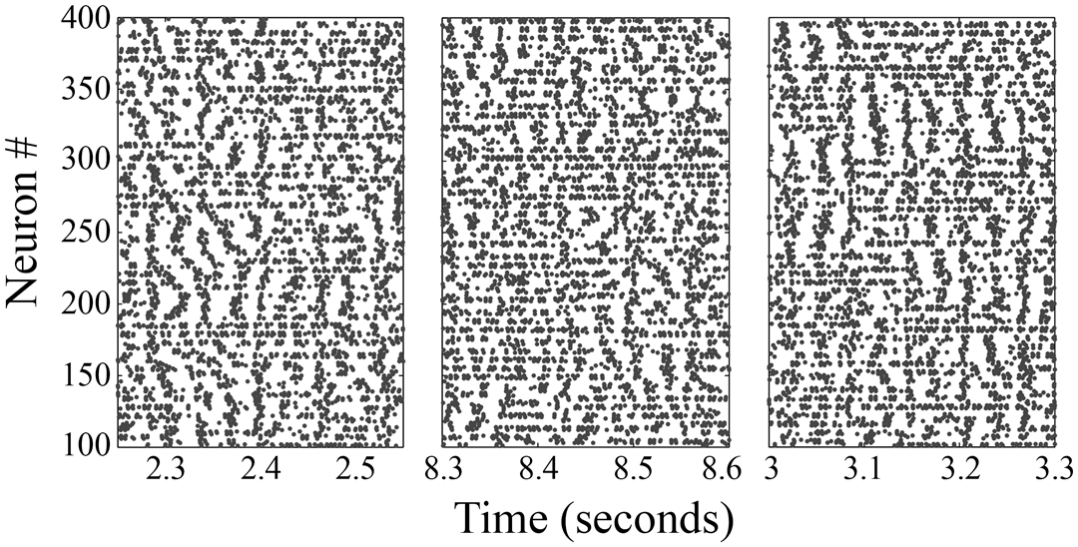
Rastergrams from the external globus pallidus of the Hahn & McIntyre model. Left) No stimulation, Middle) Closed-loop phasic burst stimulation at a phase which disrupts the 34 Hz oscillation (phase = 0.3); Right) Closed-loop phasic burst stimulation at a phase which enhances the 34 Hz oscillation (phase = 0.7).

We test closed-loop phasic stimulation and phasic burst stimulation (PhaBS) in the Hahn & McIntyre model. In this model an oscillation centered around 34 Hz emerges in the “parkinsonian state” and responds to high frequency stimulation. To implement closed-loop phasic stimulation and PhaBS in the model, a time weighted Fourier Transform of the spike trains was used to estimate the instantaneous phase of the population oscillation. This phase estimation was used to trigger stimulus pulses. First, we show that the 34 Hz pathological oscillation seen in the model can be modulated using a single stimulus pulse triggered off of the phase of the oscillation. Next, we show that applying a burst of three equally spaced stimulus pulses (5 msec apart) triggered off of the phase of the oscillation more strongly modulates the 34 Hz oscillation (raw output seen in Fig 3). Finally, we show the modulation of the 34 Hz oscillation can be predicted using the phase response curve (PRC).

**Fig 4:**
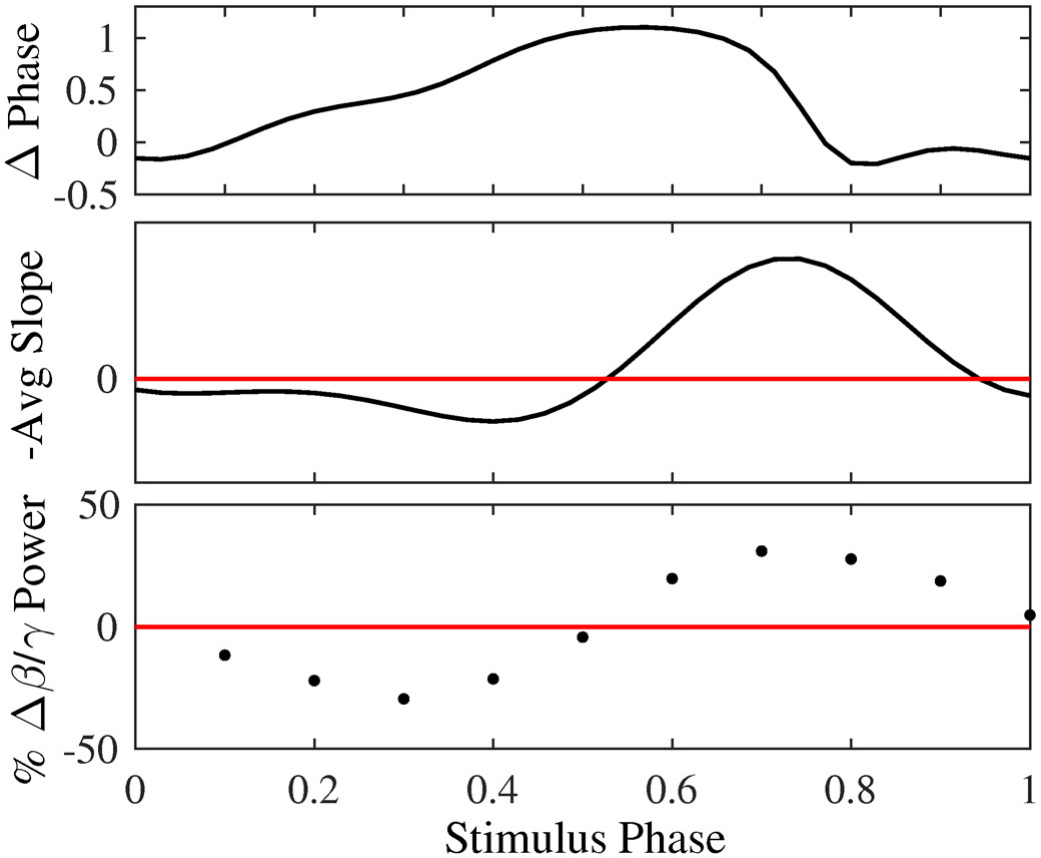
Effects of phasic stimulation with a single pulse on the population oscillation can be predicted using the PRC. Top: Population PRC, as calculated in a previous paper [20]. Middle: Single pulse predictions determined from the slope of the PRC. Prediction of which stimulus phases will desynchronize the population oscillation (below the red line) and which will enhance the oscillation (above the red line). Bottom: Ratio of Beta (31–36 Hz) to Gamma (60–64 Hz) power as a function of the stimulus phase, shown as a percent change from stimulation off condition (red line). Stimulating early in the phase suppresses the 34 Hz oscillation, while stimulating late in the phase enhances it.

Phase dependent modulation of the 34 Hz oscillation is seen using a single stimulus pulse per cycle. The ratio of the 31–36 Hz frequency band, which is modulated by stimulation, to the 60–64 Hz frequency band, which is unmodulated by stimulation for reference, shows that the stimulus phase affects the modulation of the oscillation (Fig 4). Stimulation applied early in the phase of the oscillation results in a decrease in the 34 Hz oscillation below baseline (DBS Off), while stimulation applied late in the phase of the oscillation enhances the pathological oscillation. Using 1/10th the stimulus amplitude used to model clinical DBS in the original paper [21], a 30% reduction in the beta oscillation is seen at the optimal stimulus phase.

Importantly, the PRC can be used to predict the effects of phasic stimulation on the amplitude of the 34 Hz oscillation (Fig 4). The PRC was estimated by measuring the phase advance of the oscillation as a function of the stimulus phase using a single stimulus pulse per cycle. This slope of this first order PRC can be used to predict the phase dependent modulation of the 34 Hz oscillation seen using closed-loop phasic stimulation.

**Fig 5:**
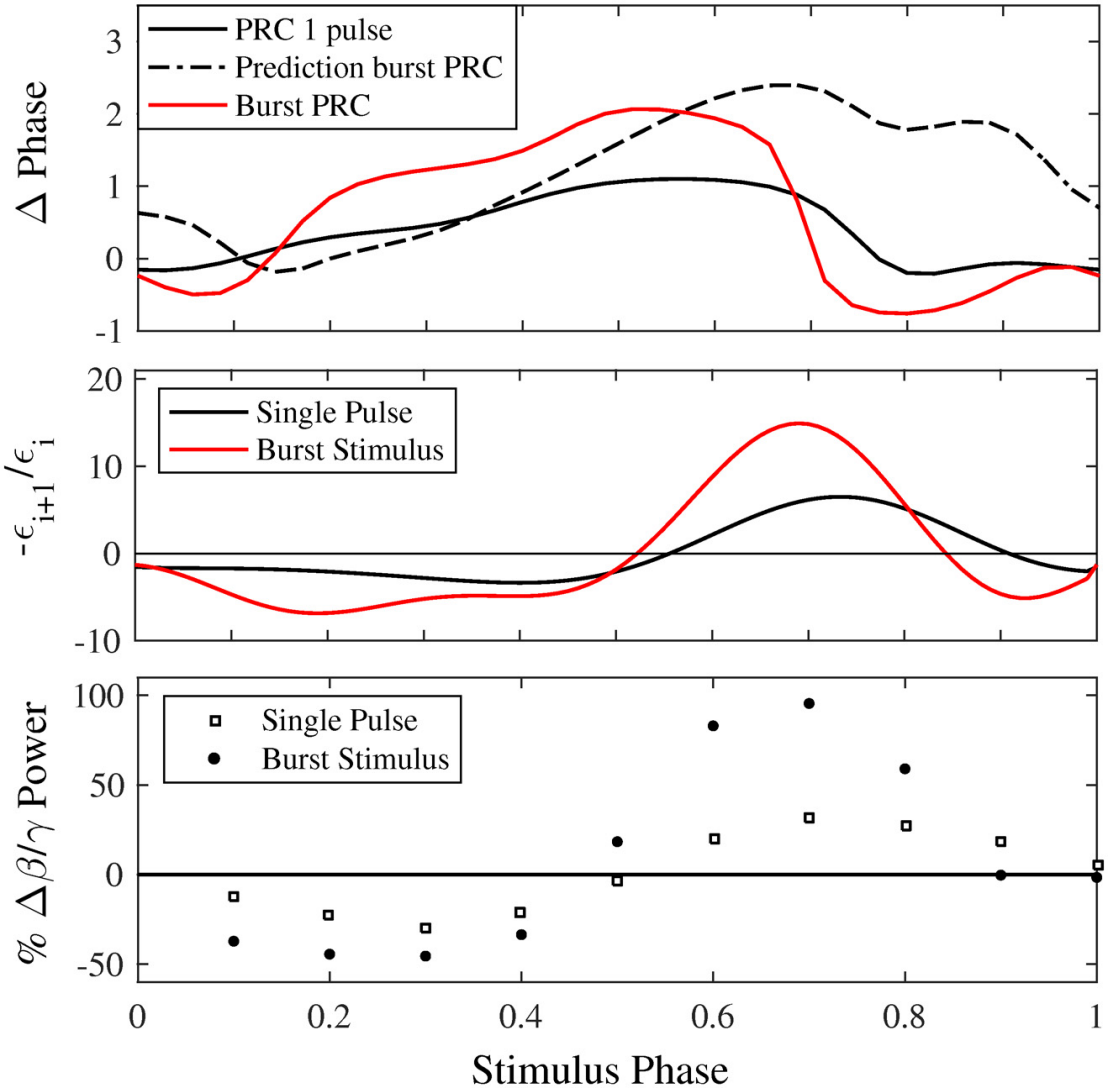
The phase response curve (PRC) can be used to predict the effects of phasic stimulation on the 34 Hz parkinsonian oscillation seen in the HM model. Top: PRCs estimated using, 1 (solid black) and 3 (red) stimulus pulses per cycle. Assuming each no interactions between pulses, the PRC for three pulses is predicted (dotted black) from the single pulse PRC. The difference seen between the prediction (dotted black) and the PRC etimated using 3 pulses (red) indicates there are higher order effects. Middle: Predictions from the single pulse PRC (black) and the 3 stimuli PRC (red). Stimulating at phases where the curve is below zero is predicted to desynchronize the oscillation. Bottom: Ratio of Beta (31–36 Hz) to Gamma (60–64 Hz) amplitude as a function of stimulus phase, shown as a percent change from stimulation off (black line at y = 0). Stimulating early in the phase suppresses the 34 Hz oscillation, while stimulating late in the phase enhances it. This matches with predictions made using the PRC (middle). Furthermore, 3 pulses per cycle result in a stronger modulation of the 34 Hz oscillation.

While a single pulse per cycle was effective at disrupting the emergent pathological oscillation, we hypothesize that a burst of stimulus pulses over a range of phases will be more effective at modulating the oscillation (Fig 2). To test phasic burst stimulation (PhaBS), a burst of three stimulus pulses triggered off the instantaneous phase was applied to the computational model. The stimulus amplitude was 1/10th the amptliude used as the clinical value in the original Hahn & McIntyre paper. Roughly 30% of the first order PRC has a positive slope (Fig 4). The inter-stimulus inverval of 5 msec was chosen so the three pulses covered about 30% of the period. As predicted in Fig 2, the effects of burst stimulation are stronger than the effects of stimulation using a single pulse per cycle. With PhaBS, almost a 50% reduction in the power of the pathological 34 Hz oscillation compared to baseline (DBS off) is seen at the optimal phase (Fig 5).

In order to use the PRC to predict effects of PhaBS on the 34 Hz oscillation, we must account for three stimulus pulses instead of a single stimulus pulse per cycle. In a first order approximation, we linearly summed the effects of each stimulus pulse on the phase advance to account for the burst stimulation. In fact, when the PRC is measured using a burst of 3 stimulus pulses, the shape does not match this first order approximation PRC (Fig 5). This indicates that there are higher order effects, and that each stimulus pulse within the burst does not result in equal phase effects. For this reason, the PRC estimated using a burst of stimui was used to predict the effects of PhaBS.

The effects of PhaBS on the 34 Hz parkinsonian oscillation seen in the Hahn & McIntyre model can be predicted using the burst PRC (Fig 5). The slope of the burst PRC is positive when stimuli are applied at phases early in the oscillation, which was found to suppress the pathological oscillation in simulations. Stimulating late in the phase, which enhances the pathological oscillation in simulations, corresponds to phases when the slope of the PRC is negative. Predictions from the PRC, plotted as the negative slope of the PRC (−*Z*′(*ϕ*_*i*_)), are shown in the middle panel of Fig 5. Negative values indicate phases predicted to desynchronize the oscillation. Furthermore, the burst PRC predicts a larger modulation of the oscillation using PhaBS than when using a single pulse, and is validated in simulations. Predictions match well with the simulation results showing the percent reduction of the pathological oscillation (Fig 5).

The PRC was used to predict the stimulus phase using Eq (3) that would produce the maximum suppression of the population oscillation. This equation can theoretically be used to optimize both the phase, *ϕ*_*i*_, and the stimulus interval, *δ*.

## Discussion

Results in this paper provide evidence for a PRC optimized closed-loop approach to DBS to suppress pathological oscillations seen in PD. This approach was tested in a computational model of the subthalamopallidal network exhibiting an emergent population oscillation in the PD state. A novel closed-loop approach to suppress oscillations, termed Phasic Burst Stimulation (PhaBS), for which the PRC is used to optimize the stimulus phase was shown to be more effective than applying a single stimulus pulse per oscillation cycle. PhaBS triggered off of the phase of pathological oscillations has the potential to improve efficacy and robustness of DBS while reducing power consumption. While the focus of this paper has been on an oscillation seen in the PD state of a computational model, the approach can be generalized to disrupt or enhance any oscillatory biomarker modulated by stimulation.

Using phasic stimulation to suppress pathological oscillations has previously been proposed and tested in the past [6, 15, 28–30]. The contribution of this paper is in providing a method for determining the optimal stimulus phase for a burst of stimulus pulses applied over a range of phases. Azodi-Avval and Gharabaghi [30] suggested using the PRC to optimize stimulation phase, but propose to stimulate at the phase of the oscillation resulting in the largest phase advance and do not test this theory. Here, we suggest that the slope of the PRC is most important in predicting phase dependent modulation of oscillatory activity, and provide numerical and theoretical evidence for this assertion.

One major advantage of PhaBS is that subthreshold amplitudes are used for stimulation. Here we have shown that applying a burst of subthreshold stimulus pulses is more effective at reducing the pathological oscillation than using a single stimulus pulse per cycle of the same amplitude. It is a possibility that using a single pulse at a higher stimulus amplitude would provide as much reduction of an oscillation as a burst of pulses. However, because the neuronal response to stimulation amplitude is highly nonlinear, we do not expect that the three small stimulus pulses can simply be replaced with one large perturbing stimulus pulse. Using a lower amplitude stimulation may reduce side effects and conserve power. Furthermore, hydrolization of the electrode is a hard safety limit of the stimulation amplitude which may be reached before significant modulation of the oscillation by a single pulse could be achieved. Therefore, if stimulation is approaching the maximum pulse amplitude, multiple pulses may be more effective.

A computational model of the subthalamopallidal network was used to test if PhaBS could desynchronize a population of heterogeneous neurons. The Hahn & McIntyre model was used because it is a well known model that produces a population oscillation which is suppressed by DBS in the PD state. The oscillation generated in this model is not caused by synchronous periodically firing neurons, but instead is an emergent property of the network generated by the coupling of an excitatory and inhibitory nucleus [20]. Previous studies have focused on applying PRC theory to single neuron oscillators [18, 31]; however here it is effectively applied to a population of neurons, as it has been done in studies of circadian oscillators [17, 32–34].

While the Hahn & McIntyre model is one of the most physiologically realistic computational models of DBS in the subthalamopallidal network, there are limitations to the predictive accuracy of this model, as described in the methods section. For computational efficacy, simplifications are made, such as with connectivity, topology, heterogeneity, and modeling the effects of stimulation on neural tissue. Despite these limitations, the Hahn & McIntyre exhibits an emergent population oscillation in the parkinsonian state and models efferent effects of stimulation, necessary for testing PhaBS. We do not suggest that the optimal phases found in this study will be the same as the optimal phase found in a clinical setting. Instead, we present evidence for a method of using the PRC, estimated empirically from each patient, to select the optimal stimulation phase to disrupt pathological oscillations specific to that subject. The use of the Hahn & McIntyre model successfully demonstrates how a closed-loop approach to DBS, where a burst of stimulus pulses is triggered off the phase of a population oscillation, may work and be systematically optimized. While here we optimize the stimulus phase, Eq (3) can also be used to optimize the inter-stimulus interval, *δ* (5 msec interval was used here).

We have proposed using PRCs to predict stimulus parameters to optimally disrupt beta oscillations seen in PD. However, there are a number of potential issues with suppressing this activity. While beta oscillations are implicated in anti-kinetic motor symptoms of PD, a causal role is highly debated [4]. Enhanced beta oscillations are reduced upon therapeutic DBS and dopamine replacement therapy [10]; however, this may be an epiphenomenon. Strong beta oscillations are not seen in all PD patients and are seen in healthy subjects, such as naïve non-human primates [35]. This suggests that beta oscillations may not be an ideal biomarker. However, the approach presented here can be be applied to any behavioral oscillation, such as tremor, or any other oscillatory activity found to be implicated in PD in the future.

It is not known how eliminating the beta oscillation will impact normal motor control. Oscillatory activity is necessary for normal function throughout the brain. Eliminating beta oscillations may impair motor control in a different way, or may allow new pathological activity to emerge. While there are many potential clinical limitations to PRC optimized PhaBS, specifically targeting beta oscillations may provide valuable insight into the role of enhanced synchrony in PD.

### Advantages of Closed-Loop DBS

Phasic Burst Stimulation, presented in this paper, is a closed-loop approach to DBS for PD. Currently continuous high frequency stimulation is used to treat motor symptoms of PD. Stimulation parameters for this open-loop approach are pre-programmed by a clinician and are not adjusted based on feedback. While high frequency stimulation is effective [36], it may not be optimal. One limitation of an open-loop approach is that the same level of stimulation is applied regardless of the severity of a patient’s motor symptoms [12]. A closed-loop approach offers many potential benefits including improved efficacy [12, 37], reduced side effects [12], increased battery life, and patient-specificity [36].

For PhaBS to be effective, an oscillatory biomarker related to symptom severity must be targeted. This oscillation could be a behavioral oscillation, such as tremor, or from a neural signal for non-oscillatory motor symptoms, such as the beta oscillation. Tremor provides a behavioral oscillation that can be recorded noninvasively from patients. It has been shown that tremor amplitude is modulated by the phase at which stimulus pulses are applied in essential tremor patients [6, 15]. The theory presented in this paper suggests that a PRC estimated from the tremor could be used to identify the optimal stimulation phase to suppress the tremor, and that a burst of stimulus pulses may be more effective than a single stimulus per cycle. An implantable DBS system for PD (Activa PC+S) with sensing, stimulation, and detection features has been developed for investigational use [2], making it possible to use the biomarkers recorded from the neural signal in a clinical setting.

In modeling, it has been shown that phasic stimulation destabilizes the current phase around which neurons synchronize while stabilizing another phase [38]. Therefore, continued stimulation at the same phase for many cycles may only result in transient destabilization and instead help sustain the oscillation after many cycles of stimulation. One solution is to turn off stimulation when the amplitude becomes small and turn it back on when the oscillation begins to emerge again.

Enhanced beta activity is not constant, there may be periods of high beta and periods of low beta synchrony [39]. For this reason, a closed-loop approach to DBS, where beta oscillations are tracked, may offer a more efficient approach to stimulation. If enhanced beta oscillations are actually causing motor symptoms, this would suggest stimulation is not needed during times when beta power is low.

To optimize PhaBS for patient-specific oscillations, a PRC must be measured from the subject. Recently it has been shown that it is possible to estimate PRCs from local field potential recordings from the STN of PD patients [30]. This suggests that the pathological beta oscillation implicated in PD is sensitive to the phase at which a stimulus is applied at that PhaBS may be possible clinically.

There have been many approaches, both closed- and open-loop, for optimizing DBS for movement disorders (i.e. [12, 28, 37, 40–44]). Adaptive and on-demand closed-loop approaches (i.e. [12, 37, 41]) have been used to reduce the amount of stimulation applied and improve efficacy. These approaches are reactive, where open-loop high frequency stimulation is applied when a physiological event is detected, such as an increase in beta power. Here we are proposing a closed-loop approach where the timing of the stimulus pulses is determined by the physiology.

New open-loop approaches, such as Coordinated Reset [42] and Temporally Optimized Patterned Stimulation (TOPS) [44] have also shown promise at improving DBS for PD. Coordinated Reset [42], a multi-site stimulation approach, aims to disrupt pathological synchrony by entraining sub-populations out of phase with each other thereby disrupting synchrony across the entire population. This approach may evoke plasticity effects resulting in long-lasting reductions in motor symptoms persistent after the stimulation is terminated. Current pulse generators implanted in patients are not capable of implementing this approach. TOPS [44], another open-loop approach, uses an algorithm to optimize the pattern of stimulation using a computational model. This approach depends on the accuracy of the computational model and is not patient-specific. PhaBS differs from these approaches by using a principled approach for optimization using a simple model, the PRC, estimated from a patient’s physiological recordings generating a patient specific stimulation.

Oscillatory activity is seen throughout the nervous system. Enhanced oscillations have been implicated in many neurological disorders, such as essential tremor, Parkinson’s disease, epilepsy, and schizophrenia [45–48], where it may be therapeutic to disrupt oscillations. However, oscillatory activity can also be necessary or important for proper function, such as such as in cognition and perception [49–52]. While this paper focuses on using PhaBS to suppress pathological oscillations seen in PD, the theory can be applied to enhance or disrupt other oscillatory signals.
